# Objective sleep characteristics and hypertension: a community-based cohort study

**DOI:** 10.3389/fcvm.2024.1336613

**Published:** 2024-03-05

**Authors:** Chunyong Chen, Bo Zhang, Jingjing Huang

**Affiliations:** ^1^ Department of Neurology, The First Affiliated Hospital of Guangxi Medical University, Nanning, Guangxi, China; ^2^ Intensive Care Medicine Department, National Hospital of Guangxi Zhuang Autonomous Region, Nanning, Guangxi, China; ^3^ Cardiac Intensive Care Unit, The First Affiliated Hospital of Guangxi Medical University, Nanning, Guangxi, China

**Keywords:** sleep, polysomnography, hypertension, non-rapid eye movement sleep, dose-response relationship

## Abstract

**Objective:**

The link between sleep quality and hypertension risk is well-established. However, research on the specific dose-relationship between objective sleep characteristics and hypertension incidence remains limited. This study aims to explore the dose-relationship association between objective sleep characteristics and hypertension incidence.

**Methods:**

A community-based prospective cohort study design was employed using data from the Sleep Heart Health Study (SHHS). A total of 2,460 individuals were included in the study, of which 780 had hypertension. Baseline personal characteristics and medical history were collected. Objective sleep characteristics were obtained through polysomnography (PSG). Multivariate logistic regression models were utilized for analysis. Restricted cubic splines (RCS) were used to examine dose-relationship associations.

**Results:**

After adjusting for covariates, the percentage of total sleep duration in stage 2 (N2%) was positively associated with hypertension incidence, while the N3% was negatively associated with hypertension incidence Odds ratio (OR) = 1.009, 95% confidence interval (CI) [1.001, 1.018], *P* = 0.037; OR = 0.987, 95% CI: [0.979, 0.995], *P* = 0.028, respectively. For every 10% increase in N2 sleep, the risk of developing hypertension increases by 9%, while a 3% decrease in N3 sleep corresponds to a 0.1% increase in the incidence of hypertension. In the subgroup of non-depression, a positive association between N2% and hypertension was significant statistically (OR = 1.012, 95%CI, 1.002, 1.021, *P* = 0.013, P_interaction_ = 0.013). RCS demonstrated that the risk of developing hypertension was lower when N2% ranged from 38% to 58% and rapidly increased thereafter (*P* = 0.002, non-linear *P* = 0.040). The lowest risk for hypertension incidence risk of N3% occurring at 25%, and a significant increase below 15% or above 40% (*P* = 0.001, non-linear *P* = 0.008).

**Conclusions:**

There's a negative association between N3% and the incidence of hypertension, and a positive association between N2% and the incidence of hypertension, particularly among non-depression individuals. These associations exhibit strong non-linear dose-response relationships.

## Introduction

Hypertension is a significant global public health issue and poses a tremendous threat to human health. It is a major risk factor for stroke and ischemic heart disease (1, 2). Over the years, researchers have been exploring the pathogenesis and influencing factors of hypertension in order to find more effective prevention and treatment methods. Sleep, as an unconventional and often overlooked risk factor for hypertension, has received increasing attention in its relationship to hypertension (3, 4). It is noteworthy that the association between sleep duration (both objectively measured and subjectively reported) and hypertension has been a topic of debate in the literature (5–10). Furthermore, certain studies have indicated that factors such as sleep efficiency, sleep quality, sleep microstructure, and fragmented sleep may also play a role in the development of hypertension (11–15), and poor subjective sleep quality assessed by the Pittsburgh Sleep Quality Index (PSQI) was demonstrated an association with the incidence of hypertension (16). Sleep stages as objective sleep characteristics in humans are divided into rapid eye movement (REM) sleep and non-rapid eye movement (NREM) sleep. NREM sleep is further categorized into N1 (stage 1), N2 (stage 2), and N3 (stages 3 and 4; also referred to as slow-wave sleep, SWS) (17). As NREM sleep progresses, sympathetic nervous system activity and blood pressure tend to decrease and recover to levels similar to wakefulness during REM sleep (18, 19). In investigations involving healthy individuals, the deprivation of SWS has been shown to significantly impede the extent of nocturnal blood pressure dipping (20). Additionally, there is evidence of a notable association between SWS and the occurrence of hypertension (21, 22), as well as a correlation between the percentage of time spent in N1, N2, or N3 sleep stages and the incidence of hypertension. Furthermore, various objective sleep characteristics, such as fragmented sleep parameters, have been linked to the prevalence of hypertension, including the arousal index in total sleep (ArI-Total), rapid eye movement sleep (ArI-REM), non-rapid eye movement sleep (ArI-NREM), fragmented sleep index (SFI), sleep efficiency (SE), and wake after sleep onset (WASO) (12). Nevertheless, there remains a gap in research regarding the dose–response relationship between these objective sleep characteristics and the incidence of hypertension.

We aim to explore the association between objective sleep characteristics and the development of hypertension, and the potentially dose-response between them. Through this study, we hope to provide evidence of the potential sleep-related influences on the development of hypertension and offer new possibilities for hypertension prevention.

## Methods

### Study design and subjects

The study design and population sample for this study were derived from the Sleep Heart Health Study (SHHS) (23, 24). SHHS is a prospective community-based cohort study designed to assess the impact of sleep-disordered breathing on cardiovascular outcomes. A total of 5,804 men and women aged 40 or older, who provided informed consent, were recruited from seven larger “parent” cohorts (referred to as SHHS1). Participants underwent a baseline examination from 1995 to 1998, which included the administration of a Sleep Habits Questionnaire, anthropometric measurements, blood pressure (BP) measurements, and overnight unattended polysomnography (PSG). After an average follow-up period of 5.3 years, the participants underwent a second examination (2001–2003), during which BP measurements were repeated. Excluded participants included individuals with hypertension at baseline (*n* = 2,478, including *n* = 102 with missing PSG data), individuals without hypertension but missing PSG data (*n* = 76), and participants with missing data, lost to follow-up, or missing hypertension records (*n* = 790). The final study cohort comprised 2,460 participants, including 780 with hypertension and 1,680 without hypertension, who were followed up longitudinally. The SHHS dataset can be accessed through the National Sleep Research Resource website (https://sleepdata.org/datasets/shhs), which provides comprehensive information about the database sources and details.

### Defining the outcome and covariates

At baseline and follow-up, participants rested for 5 min before manual blood pressure (BP) measurements were taken in a seated position, with each measurement repeated 3 times. Hypertension status is determined by the second and third blood pressure readings (Systolic BP ≥140 mm Hg and/or Diastolic BP ≥90 mm Hg) or the use of hypertension medication (25). Participant demographic characteristics, alcohol consumption, coffee and smoking history, presence of depression and/or use of antidepressant medication, medication usage information obtained through interviews, and Epworth Sleepiness Scale (ESS) were determined through questionnaires. This study considered covariates that may influence the relationship between objective sleep characteristics and the prevalence of hypertension and blood pressure. These covariates include age, sex, ethnicity, body mass index, education level, smoking, alcohol consumption, coffee intake, diabetes, chronic obstructive pulmonary disease (COPD), and depression. The participants' medical history was based on doctor's diagnosis or disclosure.

### Polysomnography

All sleep-related variables were obtained from polysomnography (PSG) data. The PSG scoring is performed in accordance with the official standards outlined in the American Academy of Sleep Medicine (AASM) scoring manual (26). Sleep duration was defined as the percentage of non-rapid eye movement (NREM) sleep (including N1, N2, and N3-4 stages, with N3-4 also defined as N3) and rapid eye movement (REM) sleep, representing the proportion of sleep time spent in each sleep stage relative to the total sleep time, and scored according to the prevailing standards at the time. Other variables obtained from PSG data include wake after sleep onset (WASO), sleep efficiency, sleep stage shifts, arousal index (number of cortical arousals per hour of sleep), AI-NREM, AI-REM, AI-All (indicating the number of respiratory abnormalities during sleep), apnea-hypopnea index (AHI, which is determined by a reduction in respiratory flow or respiratory band signal amplitude to about 25% and 70% below the baseline, respectively, for a duration exceeding 10 s) (24), and nocturnal hypoxemia (defined as the percentage of time with oxygen saturation below 90% during sleep).

### Statistical analysis

The Kruskal–Wallis test was used for continuous variables and the chi-square test was used for categorical variables to assess the differences in sample characteristics between ArI-NREM, ArI-REM, ArI-Total, Sleep duration, N1%, N2%, N3%, REM%, Sleep onset latency, Sleep efficiency, Sleep stage shifts, and WASO in relation to the occurrence of hypertension. A multivariable logistic regression model was employed, with sleep characteristic parameters as independent variables and adjusting for age, sex, race, BMI, education, smoking, alcohol consumption, coffee intake, total cholesterol, diabetes, COPD, benzodiazepine use, depression, antidepressant use, apnea-hypopnea index (AHI), oxygen saturation below 90% (SO2 < 90%), and ESS questionnaire score as covariates to analyze the dependent variable, hypertension. Model 1 represents the unadjusted model; Model 2 adjusts for age and sex; and Model 3 adjusts for age, sex, race, BMI, education, smoking, alcohol consumption, coffee intake, total cholesterol, diabetes, COPD, benzodiazepine use, depression, antidepressant use, AHI, SO2 < 90%, and ESS. To account for potential nonlinear associations between objective sleep characteristics and the development of hypertension, restricted cubic spline (RCS) plots were computed and visualized using R Studio 4.0 software.

## Results

**Figure 1:**
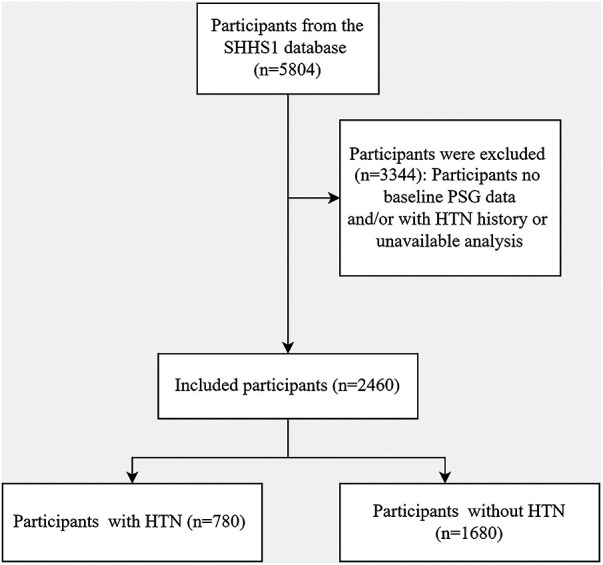
A flowchart illustrating the participant selection process in the sleep heart health study (SHHS) visit1. Out of the initial 5,804 participants, 2,460 participants were included in the final analysis. HTN, Hypertension; PSG, Polysomnography.

In the Sleep Heart Health Study phase 1 (SHHS1), the initial cohort consisted of 5,804 participants. The flow chart (Figure 1) delineates the selection process, identifying 2,460 participants who lacked hypertension at baseline, completed a full polysomnography (PSG) session successfully, and had their hypertension status or antihypertensive medication use verified during the follow-up. Over an average follow-up period of 5.3 ± 0.5 years, hypertension was newly diagnosed in 780 participants, while 1,680 participants did not develop hypertension.

The differences between the hypertension and non-hypertension groups are showed (Table 1). The hypertension group, compared to the non-hypertension group, exhibited higher average age (64.5 vs. 59.0 years, *P* < 0.001), lower proportion of females (51.7% vs. 56.1%, *P* = 0.045), higher BMI (28.6 vs. 27.3, *P* < 0.001), lower level of education (*P* = 0.023), higher smoking rate (46.3% vs. 41.0%, *P* = 0.038), and higher prevalence of diabetes (5.8% vs. 2.2%, *P* < 0.001). Additionally, the hypertension group had a higher apnea-hypopnea index (AHI) (18.3 vs. 15.0, *P* < 0.001) and longer duration of nighttime oxygen saturation <90% (3.3% vs. 2.1%, *P* < 0.001) at baseline. Follow-up data revealed significantly higher systolic blood pressure (135 vs. 120 mmHg, *P* < 0.001) and diastolic blood pressure (72 vs. 70 mmHg, *P* < 0.001) in the hypertension group. Baseline PSG indicated the hypertension group showed a higher NREM respiratory event index, total respiratory event index, shorter sleep duration, higher N2%, lower N3%, lower sleep efficiency, and longer wake after sleep onset (WASO) time.

**Table 1:** Characteristics in participants with or without hypertension.

Variables	All (*n* = 2,460)	Hypertension (*n* = 780)	Non-hypertension (*n* = 1,680)	*P*-value
Age, year	60.7 (10.4)	64.5 (10.0)	59.0 (10.1)	<0.001
Sex, *n* (%)				
Women	1,345 (54.7)	403 (51.7)	942 (56.1)	0.045
Race, *n* (%)				0.002
White	2,188 (88.9)	698 (89.5)	1,490 (88.7)	
African American	100 (4.1)	43 (5.5)	57 (3.4)	
Other	172 (7.0)	39 (5.0)	133 (7.9)	
BMI	27.7 (4.7)	28.6 (4.9)	27.3 (4.5)	<0.001
Education, *n* (%)				0.023
<10 years	171 (7.0)	66 (8.5)	105 (6.3)	
11-15 years	1,275 (51.8)	418 (53.6)	857 (51)	
16-20 years	872 (35.4)	262 (33.6)	610 (36.3)	
> 20 years	142 (5.8)	34 (4.4)	108 (6.4)	
Smoking status, *n* (%)				0.038
Current smoker	263 (10.7)	82 (10.5)	181 (10.8)	
Former smoker	1,049 (42.6)	361 (46.3)	688 (41.0)	
Alcohol, *n* (%)	1,113 (45.2)	354 (45.4)	759 (45.2)	0.931
Coffee, *n* (%)	1,566 (63.7)	488 (62.6)	1,078 (64.2)	0.444
Benzodiazepine use, *n* (%)	85 (3.5)	31 (4.0)	54 (3.2)	0.344
TCA, NTCA use, *n* (%)	157 (6.4)	58 (7.4)	99 (5.9)	0.156
Depression, *n* (%)	225 (9.1)	80 (10.3)	145 (8.6)	0.202
Diabetes Mellitus, *n* (%)	82 (3.3)	45 (5.8)	37 (2.2)	<0.001
COPD, *n* (%)	16 (0.4)	9 (1.2)	7 (0.4)	0.055
Epworth Sleepiness Scale	7.6 (4.2)	7.7 (4.1)	7.6 (4.2)	0.697
AHI	16.0 (14.2)	18.3 (16.0)	15.0 (13.2)	<0.001
SaO2 < 90%	2.5 (7.7)	3.3 (9.3)	2.1 (6.9)	<0.001
Sleep characteristics				
ArI-NREM, events/h	19.2 (10.5)	20.6 (11.7)	18.5 (9.8)	<0.001
ArI-REM, events/h	15.0 (10.2)	15.2 (10.8)	14.9 (9.9)	0.553
ArI-Total, events/h	18.4 (9.6)	19.6 (10.7)	17.8 (9.0)	<0.001
Sleep duration (min)	372.6 (58.7)	363.8 (62.2)	376.7 (56.6)	<0.001
N1 (%, TST)	5.2 (3.6)	5.3 (3.7)	5.1 (3.6)	0.109
N2 (%, TST)	56.2 (11.2)	57.4 (11.6)	55.6 (10.9)	<0.001
N3 (%, TST)	18.0 (11.3)	16.9 (11.6)	18.5 (11.1)	0.001
REM (%,TST)	20.7 (5.8)	20.3 (5.8)	20.8 (5.8)	0.060
Sleep onset latancy (min)	12.8 (19.0)	13.8 (20.0)	12.4 (18.6)	0.082
Sleep efficiency (%)	84.8 (9.1)	83.5 (9.6)	85.4 (8.8)	<0.001
Sleep stages shifts	27.5 (12.3)	28.0 (12.7)	27.3 (12.2)	0.205
WASO (min)	54.9 (39.1)	59.1 (41.5)	53.0 (37.8)	<0.001
SBP/mmHg	125 (15.0)	135 (17.9)	120 (10.5)	<0.001
DBP/mmHg	70 (9.1)	72 (11.0)	70 (8.0)	<0.001

AHI, apnea hypopnea index; ArI, arousal index; BMI, body mass index; COPD, chronic obstructive pulmonary disease; NREM, non-rapid eye movement; NTCA, non-tricylic anti-depressants; REM, rapid eye movement; SE, sleep efficiency; TCA, tricylic anti-depressants; TST, total sleep time; WASO, wake after sleep onset; y, years.

Results are presented as mean (standard deviation) or *n* (%). The *P*-values represent the difference between or among groups.

The findings of this study revealed significant associations between specific parameters in PSG and the incidence of hypertension (Table 2). In Model 1, the arousal index (ArI-NREM and ArI-Total) demonstrated a positive correlation with hypertension onset (OR = 1.017, 95% CI, 1.010, 1.025, *P* < 0.001; and OR = 1.017, 95% CI, 1.009, 1.025, *P* < 0.001, respectively). These associations remained statistically significant in Model 2 (OR = 1.009, 95% CI, 1.001, 1.017, *P* = 0.026; and OR = 1.009, 95% CI, 1.000, 1.017, *P* = 0.048, respectively). However, these associations became non-significant in Model 3 (all *P* > 0.05). Sleep duration and sleep efficiency exhibited a negative correlation with hypertension incidence in Model 1 (OR = 0.998, 95% CI, 0.997, 0.999, *P* = 0.001; and OR = 0.984, 95% CI, 0.976, 0.993, *P* < 0.001, respectively). Additionally, WASO showed a positive relationship with hypertension in model 1 (OR = 1.003, 95% CI, 1.001, 1.005, *P* = 0.002), but it did not remain statistically significant onset in subsequent models. The N2% and N3% showed a positive and negative correlation with hypertension onset, respectively, in Model 1 (OR = 1.012, 95% CI, 1.005, 1.020, *P* = 0.001; and OR = 0.988, 95% CI, 0.981, 0.995, *P* = 0.001, respectively) and Model 2 (OR = 1.009, 95% CI, 1.002, 1.017, *P* = 0.019; and OR = 0.988, 95% CI, 0.980, 0.995, *P* = 0.002, respectively). These associations remained significant in Model 3 (OR = 1.009, 95% CI, 1.001, 1.018, *P* = 0.037; and OR = 0.987, 95% CI, 0.979, 0.995, *P* = 0.028, respectively).

**Table 2:** Models of odds ratios (ORs) and the 95% confidence intervals for PSG associated with hypertension incidence.

PSG	Model 1	Model 2	Model 3
ArI-NREM, events/h	1.017 (1.010, 1.025)***	1.009 (1.001, 1.017)*	1.007 (0.997, 1.017)
ArI-REM, events/h	1.001 (0.993, 1.009)	1.000 (0.992, 1.008)	0.996 (0.987, 1.005)
ArI-Total, events/h	1.017 (1.009, 1.025)***	1.009 (1.000, 1.017)*	1.006 (0.995, 1.017)
Sleep duration (min)	0.998 (0.997, 0.999)**	0.999 (0.998, 1.001)	0.999 (0.998, 1.001)
N1 (%, TST)	1.013 (0.991, 1.035)	0.996 (0.973, 1.019)	0.991 (0.968, 1.015)
N2 (%, TST)	1.012 (1.005, 1.020)**	1.009 (1.002, 1.017)*	1.009 (1.001, 1.018)*
N3 (%, TST)	0.988 (0.981, 0.995)**	0.988 (0.980, 0.995)**	0.987 (0.979, 0.995)**
REM (%,TST)	0.997 (0.983, 1.010)	1.012 (0.997, 1.026)	1.016 (1.002, 1.031)*
Sleep onset latency (min)	1.003 (0.999, 1.007)	1.004 (0.999, 1.008)	1.004 (0.999, 1.008)
Sleep efficiency (%)	0.984 (0.976, 0.993)***	0.998 (0.989, 1.007)	0.999 (0.990, 1.008)
Sleep stages shifts	1.004 (0.998, 1.011)	0.999 (0.992, 1.006)	0.998 (0.991, 1.005)
WASO (min)	1.003 (1.001, 1.005)**	0.999 (0.997, 1.001)	0.999 (0.997, 1.001)

min, minutes; y, years old; w, week; AHI, apnea hypopnea index; ArI, arousal index; BMI, body mass index; COPD, chronic obstructive pulmonary disease; NREM, non-rapid eye movement; NTCA, non-tricylic anti-depressants; PSG, polysomnography; REM, rapid eye movement; SE, sleep efficiency; TCA, tricylic anti-depressants; TST, total sleep time; WASO, wake after sleep onset; y, years.

Model 1 unvariable model.Model 2 adjusted for age and sex. Model3 adjusted for age, sex, race, BMI, education, smoking, alcohol consumption, coffee intake, total cholesterol, diabetes, COPD, benzodiazepine use, depression, antidepressant use, apnea-hypopnea index (AHI), and oxygen saturation below 90% (SO2 < 90%), and ESS (Epworth Sleepiness Scale).

* *P* < 0.05.

** *P* < 0.01.

*** *P* < 0.001.

**Figure 2:**
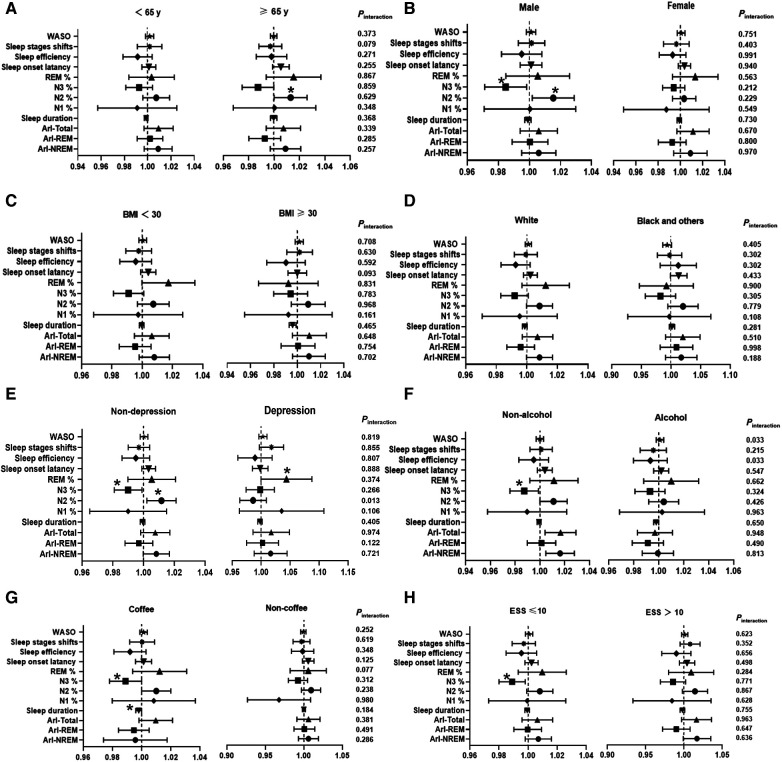
Subgroup analysis investigating the association between sleep characteristic biomarkers and the incidence of hypertension. The results are presented as multivariable-adjusted odds ratios with covariate adjustments for age, sex, race, body mass index, education, smoking, alcohol consumption, coffee intake, total cholesterol, diabetes, chronic obstructive pulmonary disease (COPD), benzodiazepine use, depression, antidepressant use, apnea-hypopnea index (AHI), oxygen saturation below 90% (SO2 < 90%), ESS, and Epworth Sleepiness Scale. NREM, Non-rapid eye movement sleep; REM, Rapid eye movement sleep; ArI-Total, Arousal Index—Arousals per hour of sleep; ArI-REM, Arousal Index in REM; ArI-NREM, Arousal Index in NREM; N1%, Percentage of total sleep duration in stage 1; N2%, Percentage of total sleep duration in stage 2; N3%, Percentage of total sleep duration in stage 3−4; WASO, Wake after sleep onset. *P < 0.05, **P < 0.01.

The forest plot in Figure2 showed a significant positive association between N2 sleep stage percentage and the incidence of hypertension in subgroups of older individuals (≥65 years), males, and individuals without depression (≥65 years: OR = 1.013, 95% CI, 1.000, 1.026, *P* = 0.049, P_interaction_ = 0.859, Figure 2A; male: OR = 1.015, 95% CI, 1.002, 1.029, *P* = 0.024, P_interaction_ = 0.212, Figure 2B; non-depression: OR = 1.012, 95% CI, 1.002, 1.021, *P* = 0.013, P_interaction_ = 0.013, Figure 2E). In the subgroup of male, non-depression individuals, non-drinkers, individuals with non-coffee intake, and those with an Epworth Sleepiness Scale (ESS) score ≤10, there was a stronger negative correlation between N3 sleep stage percentage and the occurrence of hypertension (male: OR = 0.985, 95% CI, 0.971, 0.998, *P* = 0.029, P_interaction_ = 0.229, Figure 2B; non-depression: OR = 0.990, 95% CI, 0.981, 0.999, *P* = 0.035, _Pinteraction_ = 0.226, Figure 2E; non-drinkers: OR = 0.987, 95% CI, 0.976, 0.999, *P* = 0.031, P_interaction_ = 0.285, Figure 2F; non-coffee intake: OR = 0.989, 95% CI, 0.978, 1.000, *P* = 0.046, P_interaction_ = 0.312, Figure 2G; ESS score ≤10: OR = 0.990, 95% CI, 0.980, 1.000, *P* = 0.043, P_interaction_ = 0.867, Figure 2H). As described above, among all the twelve objective sleep characteristic markers for hypertension risk, only N2% was modulated by depression.

**Figure 3:**
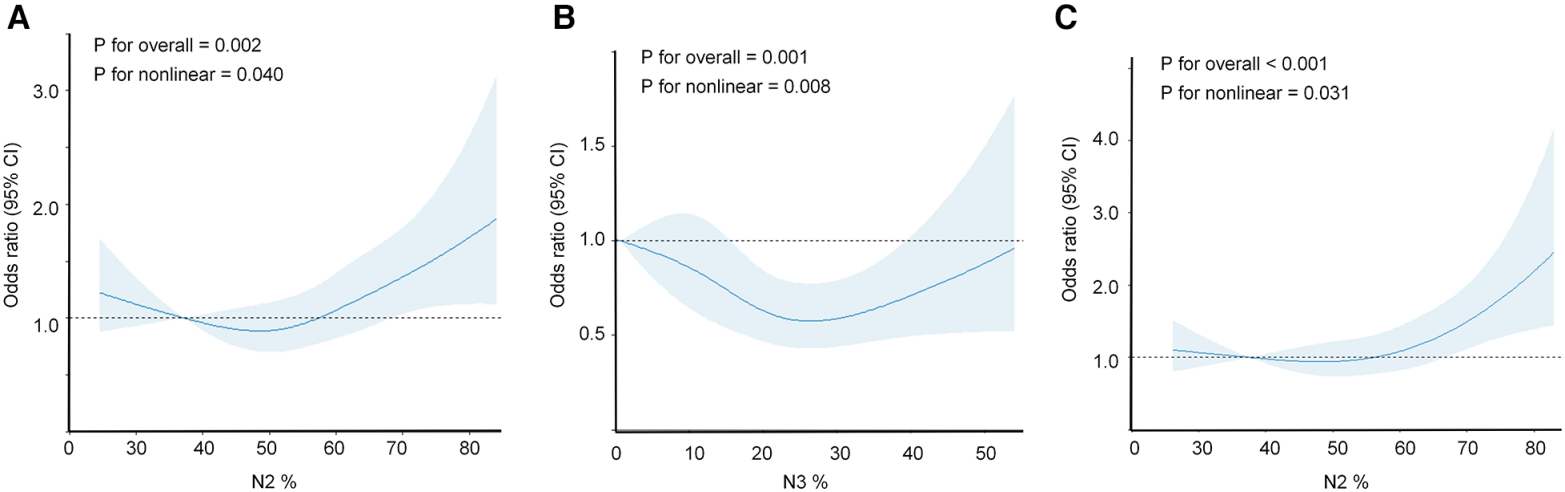
Restricted cubic spline regression analysis illustrating the association between objective sleep characteristics and incidence of hypertension. The association between N2% or N3% and hypertension incidence was assessed using a restricted cubic spline regression model. The graphs present odds ratios (ORs) for hypertension incidence based on N2% (**A**) or N3% (**B**), or in the subgroup of non-depression individuals (**C**) The models were adjusted for age, sex, race, BMI, education, smoking, alcohol consumption, coffee intake, diabetes, COPD, benzodiazepine use, depression, antidepressant use, AHI, and SO2 < 90%. The data were fitted using a logistic regression model, with four knots placed at the 5th, 35th, 65th, and 95th percentiles of N2%, N3%, or the non-depression subgroup (the reference being the 5th percentile). The solid lines represent the odds ratios, and the shaded areas indicate the 95% confidence intervals. OR, odds ratio; CI, confidence interval; BMI, body mass index; COPD, chronic obstructive pulmonary disease; AHI, apnea-hypopnea index; SO2 < 90%, oxygen saturation below 90%.

Furthermore, we employed restricted cubic splines (RCS) to flexibly model and visualize the relationship between N2% (Figure 3A) and N3% (Figure 3B) with the incidence of hypertension in all participants. The risk of hypertension incidence was relatively low between 38% and 58% of N2% and then exhibited a rapid increase thereafter (*P* for overall = 0.002, *P* for non-linearity = 0.040). Regarding the strong U-shaped relationship between N3% and hypertension incidence, the plot demonstrated a significant reduction in risk within the lower range of N3%. The lowest risk of hypertension incidence was observed at 25% of N3, with a relatively lower risk between 15% and 40%; however, the risk of hypertension incidence showed a noticeable increase for values below 15% or above 40% (*P* for overall = 0.001, *P* for non-linearity = 0.008). In the non-depression subgroup (Figure 3C), the risk of hypertension incidence remained relatively low and stable around 58% of N2, and then demonstrated a rapid increase thereafter (*P* for overall <0.001, *P* for non-linearity = 0.031).

## Discussion

This large-scale cohort study conducted on middle-aged and older women and men revealed that a less N3% or a more N2% were associated with an increased risk of developing hypertension. The study also found significant differences between the hypertension and non-hypertension groups in various baseline demographic and objective sleep characteristics. Specifically, the hypertension group had a higher average age, lower proportion of female, higher BMI, lower education level, higher smoking rates, and a higher prevalence of diabetes. These findings are consistent with existing research and lend support to the notion that these factors may have a certain impact on the development of hypertension (27, 28).

In terms of sleep hypoxia characteristics, the hypertension group showed higher apnea-hypopnea index (AHI) and spent a longer duration with nighttime blood oxygen saturation below 90%. These findings suggest that sleep-disordered breathing is associated with the development of hypertension, which is consistent with previous research (29). Polysomnography (PSG) provides more objective evidence regarding sleep characteristics. Our study found that changes in objective sleep characteristics recorded by PSG are associated with the development of hypertension. The hypertension group exhibited a higher index of wakefulness during NREM sleep, higher overall arousal index, shorter sleep duration, higher N2%, lower N3%, lower sleep efficiency, and longer wake after sleep onset (WASO) at baseline. These results indicate that hypertension patients already exhibit certain abnormalities in their sleep patterns several years prior to the onset of hypertension, which may be related to the development of hypertension. Our findings align with previous research, particularly in the negative correlation between N3 sleep percentage and the development of hypertension (21). Our study also found that arousal index (ArI-NREM and ArI-Total) showed a positive correlation with hypertension in Model 1 and Model 2. However, in Model 3 (after adjusting for all potential confounding factors), this association was no longer significant, suggesting that the relationship between arousal index and hypertension may be influenced by other factors. For instance, previous research has shown that the degree of hypoxia acts as a confounding factor in the association between arousal index and the development of hypertension (30). These findings are consistent with other studies, indicating that an increased arousal index suggests an increase in sleep fragmentation, which may raise the risk of hypertension (31, 32). Frequent awakenings disrupt sleep continuity, hinder the achievement and maintenance of deep sleep, and consequently result in poor sleep quality and daytime fatigue (33, 34).

In this study, we observed objective sleep characteristics associated with the development of hypertension in specific subgroups. For older individuals (≥65 years), male, and non-depression individuals, we found a stronger positive correlation between the N2% and hypertension. In the subgroup of male, non-depression individuals, non-drinkers, non-coffee consumers, and individuals with an Epworth Sleepiness Scale (ESS) score ≤10, we observed a stronger negative correlation between the percentage of N3 sleep stage and hypertension. After adjusting for all potential confounding factors, our study found a complex non-linear relationship between N2% and N3% and hypertension. This suggests that changes in sleep stage may influence the risk of developing hypertension, but this influence may vary within different ranges of sleep stage percentages. In adults, there is a normal range for the proportion of sleep stages during the total sleep duration. N1% accounts for 2%–5%, N2% accounts for 45%–55%, N3% accounts for 13%–23%, and REM % accounts for 20%–25% (35). Previous studies have also shown that the percentage of N3 slow-wave sleep in NREM sleep may be the risk of hypertension, with a negative correlation observed (22). Conversely, the N2% is positively correlated with the risk of hypertension (21), and the lowest risk of hypertension occurs when N3% is between 17% and 25% (22). In our study, If N2 sleep increases by 10%, the risk of developing hypertension increases by 9%, whereas a 3% reduction in N3 sleep is associated with a 0.1% rise in the incidence of hypertension. Furthermore, in our study, we found a non-linear relationship between the N3% and N2% variables and the risk of hypertension. Therefore, we used restricted cubic splines (RCS) to optimize the statistical analysis for non-linear variables. The results showed that the lowest risk of hypertension incidence occurs at 25% of N3%, and the relative risk of hypertension is lower between 15% and 40% of N3%. However, there is a significant increase in the risk of hypertension incidence when N3% is below 15% or above 40%. Additionally, the high percent of light sleep N2 also has an important role in the risk of hypertension incidence. The risk of hypertension incidence is relatively low between 38% and 58% of N2%, and then it starts to increase rapidly afterwards. Prolonging deep sleep N3 is considered beneficial, a recent study utilized non-phase-locked pink noise stimulation to prolong N3 sleep and improve sleep quality (36). In our study, the RCS was utilized to optimize the statistical analysis for non-linear variables, providing a robust method for capturing the intricate relationship between the percents of sleep stages and hypertension incidence. This approach has allowed us to identify critical thresholds for N3% and N2% that are associated with the lowest risk of hypertension incidence, as well as the subsequent escalation of risk beyond these thresholds. To our knowledge, previously studies did not provide these relationships. We believe that our findings have the potential to advance the understanding of sleep-related risk factors for hypertension and contribute to the development of targeted interventions for at-risk populations.

The animal study indicated that sleep deprivation could heighten sympathetic nervous activity, potentially precipitating high blood pressure (37, 38). The underlying mechanism, characterized by inflammation, oxidative stress, and endothelial dysfunction, marks a pathological state in insomnia, whereas hypertension can disrupt melatonin secretion, alter circadian rhythms, and precipitate sleep disorders (39, 40). As sleep deepens, such as entering the N3 sleep stage, sympathetic nerve activity decreases, parasympathetic nerve activity increases, and baroreflex sensitivity also enhances, which leads to decreased blood pressure and heart rate. In contrast, if N3 sleep is reduced and N2 sleep increases, blood pressure and heart rate would increase under the excitation of excessive sympathetic activity (41). Another studies suggested that abnormal alternations in sympathetic-parasympathetic nervous system excitation affected by hypothalamic-pituitary-adrenal axis can cause vascular damage, the nighttime elevation of blood pressure due to increased sympathetic excitation may be associated with daytime chronic hypertension (42, 43). The aforementioned mechanisms could provide suitable explanations for the association between sleep stage proportions and hypertension in this study. Furthermore, future research should prioritize investigating the mechanisms that contribute to their interaction.

To date, the causal mechanisms linking sleep characteristics or disorders with hypertension have not been elucidated. Clinical research has revealed that shorter objective sleep duration or reduced sleep maintenance is associated with an increase in both systolic and diastolic blood pressure over a follow-up period of five years (44). Recent studies using Mendelian Randomization have shown the risk of hypertension is elevated by sleep apnea and snoring, with BMI potentially playing a role in driving these associations (45). Additionally, both short and long sleep durations, as well as disruptions in circadian rhythms, are associated with a higher likelihood of developing hypertension (46). Existing studies have demonstrated that, compared to individuals without Obstructive Sleep Apnea (OSA), patients with OSA have a significantly higher risk of developing hypertension, moreover, the risk of developing hypertension significantly escalates following the treatment for OSA, indicating that OSA is a significant risk factor for the onset of hypertension (47). However, it's worth noting that not only can sleep disorders increase the incidence of hypertension, but the reverse may also be true. Previously study had conducted a bidirectional Meta-Analysis of prospective cohort studies and discovered a statistically significant bidirectional correlation between insomnia and hypertension (48). These studies indicate that the causal link between sleep and high blood pressure warrants further investigation.

Interestingly, we observed a persistent positive correlation between the N2% and the risk of hypertension in the non-depression subgroup. Among the twelve objective sleep characteristics related to hypertension risk, only the N2% was modulated by the presence of depression, and this association was statistically significant. Further RCS analysis revealed that the risk of hypertension incidence remained relatively flat within approximately 58% of N2% and then increased rapidly thereafter. These correlations were not significant in the depression population. These findings suggest a complex relationship between the percentages of different sleep stages and the risk of hypertension, which may be influenced by an individual's depressive state. A recent cross-sectional study demonstrated a synergistic interaction between depression, sleep disorders, and hypertension (49). Previous study has shown that depression or hypertension is independently associated with subjective sleep quality (50). Other research has confirmed the independent associations of depression or sleep disorders and hypertension (51). Based on these findings, it is speculated that in the present study, objective sleep characteristics in non-depressed individuals is associated with the incidence of hypertension. However, this linking value does not appear to be evident in depressed individuals, possibly due to the independent influence of depression on sleep disorders and hypertension. Although the underlying mechanisms have not been fully elucidated and warrant further investigation.

It should be noted that our study has several limitations. Firstly, the results of this study are based solely on observational research, and although the findings suggest that baseline PSG objective sleep characteristics have an important role for the development of hypertension, they do not establish a causal relationship between sleep features and hypertension. Further research is still needed to determine the specific relationship between sleep quality and hypertension and to explore the impact of improving sleep quality on the prevention and treatment of hypertension. Secondly, our study sample primarily consisted of a large community cohort and may not be representative of all populations. Furthermore, the baseline hypertension data in this study was collected solely through questionnaire surveys to determine the presence or absence of hypertension. However, at the time of data collection, the diagnostic criteria for hypertension were defined as systolic blood pressure (SBP) ≥ 140 mmHg and/or diastolic blood pressure (DBP) ≥ 90 mmHg. The study did not incorporate the updated diagnostic criteria from the 2017 American College of Cardiology/American Heart Association guidelines, which define hypertension as SBP ≥ 130 mmHg and/or DBP ≥ 80 mmHg. The diagnosis of hypertension has traditionally relied solely on office blood pressure measurements. However, we acknowledge that for an accurate diagnosis of high blood pressure, it is necessary to conduct 24-hour ambulatory blood pressure monitoring (ABPM) (25). ABPM enables the collection of crucial data on blood pressure changes associated with sleep. Moreover, there is currently a lack of data regarding the presence of other significant sleep disorders, such as insomnia and restless legs syndrome, which may have the potential to impact nighttime sleep-related blood pressure values (52). We acknowledge the importance of investigating the potential effects of these disorders on NREM sleep stages and their potential relationship to hypertension. Lastly, our study did not account for all possible confounding factors, such as work status, lifestyle factors and genetic factors. Future research is needed to further investigate the influence of these factors on the sleep-hypertension relationship.

## Conclusion

In conclusion, this study highlights that there's complex relationship between objective sleep characteristics and hypertension. Different sleep parameters may have varying associations with the risk of hypertension. For example, an increase in N2% may elevate the risk of hypertension, while an increase in N3% within a certain range may potentially lower the risk of hypertension. With a 10% increase in N2 sleep, there is a 9% higher risk of developing hypertension, while a 3% decrease in N3 sleep is linked to a 0.1% increase in the incidence of hypertension. Dose-response analysis showed the risk of hypertension incidence was relatively low between 38% and 58% of N2%, and then it started to increase rapidly afterwards. There was a significant increase in the risk of hypertension incidence when N3% is below 15% or above 40%.

## Data Availability

The original contributions presented in the study are included in the article/Supplementary Materials, further inquiries can be directed to the corresponding author.
